# Quantitative and Qualitative Analyses of the Cell Death Process in *Candida albicans* Treated by Antifungal Agents

**DOI:** 10.1371/journal.pone.0028176

**Published:** 2011-12-09

**Authors:** Kyung Sook Kim, Young-Sun Kim, Ihn Han, Mi-Hyun Kim, Min Hyung Jung, Hun-Kuk Park

**Affiliations:** 1 Department of Biomedical Engineering, College of Medicine, Kyung Hee University, Seoul, Korea; 2 Healthcare Industry Research Institute, Kyung Hee University, Seoul, Korea; 3 Department of Obstetrics and Gynecology, School of Medicine, Kyung Hee University, Kyung Hee University Hospital, Seoul, Korea; 4 College of Oriental Medicine, Kyung Hee University, Seoul, Korea; 5 Program of Medical Engineering, Kyung Hee University, Seoul, Korea; University of Massachusetts Medical, United States of America

## Abstract

The death process of *Candida albicans* was investigated after treatment with the antifungal agents flucytosine and amphotericin B by assessing morphological and biophysical properties associated with cell death. *C. albicans* was treated varying time periods (from 6 to 48 hours) and examined by scanning electron microscopy (SEM) and atomic force microscopy (AFM). SEM and AFM images clearly showed changes in morphology and biophysical properties. After drug treatment, the membrane of *C. albicans* was perforated, deformed, and shrunken. Compared to the control, *C. albicans* treated with flucytosine was softer and initially showed a greater adhesive force. Conversely, *C. albicans* treated with amphotericin B was harder and had a lower adhesive force. In both cases, the surface roughness increased as the treatment time increased. The relationships between morphological changes and the drugs were observed by AFM clearly; the surface of *C. albicans* treated with flucytosine underwent membrane collapse, expansion of holes, and shrinkage, while the membranes of cells treated with amphotericin B peeled off. According to these observations, the death process of *C. albicans* was divided into 4 phases, CDP_0_, CDP_1_, CDP_2_, and CDP_4_, which were determined based on morphological changes. Our results could be employed to further investigate the antifungal activity of compounds derived from natural sources.

## Introduction

*Candida albicans* (*C. albicans*) is a dimorphic organism that forms either a yeast or a mycelium depending on the growth environment. *C. albicans* is normally present on the skin and in mucous membranes such as the vagina, mouth, and rectum in 80% of the human population with no harmful effects; overgrowth results in candidiasis, which is generally easily cured [1]–[5]. However, candidiasis can sometimes result in serious opportunistic infections when the immune system is compromised, and is an important cause of morbidity and mortality in immunocompromised patients with diseases such the human immunodeficiency virus (HIV), or undergoing chemotherapy, organ or bone marrow transplantation. Mortality associated with candidiasis can be as high as 30–50% in patients undergoing transplantation and 40% of those with HIV [5].

The current antifungal agents available to treat candidiasis can be divided into two groups depending on mechanism of action [6]. One group acts mainly on cell walls or membranes, while the other attacks intracellular pathways. The antifungal agents of the former group are typically azole-type drugs, which are ergosterol inhibitors or b-glucagon synthase inhibitors [6]. Those in the latter group include pyrimidine analogues, thymidylate synthase inhibitors, and mitotic inhibitors [6]. However, treatment of *C. albicans* infections with these drugs may lead to side effects such as hepatotoxicity and nephrotoxicity [7], resistance, and frequent recurrence. For these reasons, treatment using home remedies has been widely introduced worldwide, and new antifungal agents from natural resources are being studied intensively. Several natural compounds show antifungal activity, including *Euphorbia hirta* L [3], *Eqoul*, which is found in soybean [4], *Tribulus terrestris* L [8], and *Allicin*, which is found in garlic [7]. Despite several reports describing drugs extracted from natural resources, thus far, very little is known about their antifungal activities.

In this work, we demonstrate the death process of *C. albicans* after the use of two commonly used antifungal agents, flucytosine and amphotericin B. Both have been used in the treatment of candidiasis, and their mechanisms of actions are well known. We investigated changes in morphology and biophysical properties of *C. albicans* treated with flucytosine and amphotericin B using scanning electron microscopy (SEM) and atomic force microscopy (AFM). The findings of this work may be used in future studies to investigate the antifungal activities of natural drugs.

## Results

**Figure 1:**
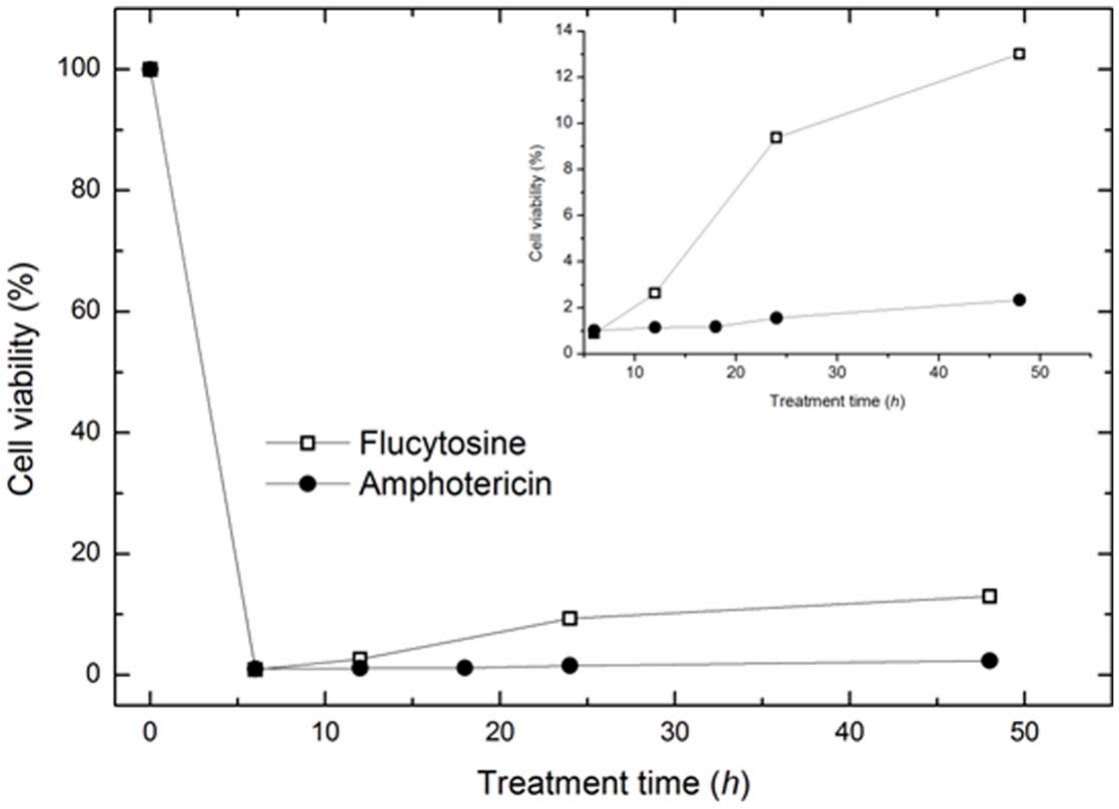
Cell viability of *C. albicans* as a function of antifungal drug treatment time. The cells were treated with two commonly used antifungal drugs, flucytosine and amphotericin B.

The antifungal activities of flucytosine and amphotericin B were estimated using a cell viability assay. The rates of *C. albicans* viability as a function of drug treatment time from 0 (control) to 48 hours are shown in Figure 1. The inset shows results for treatment times from *h* = 6 to *h* = 48. When *C. albicans* was exposed to either antifungal drugs for *h* = 6, the viability decreased by 90%. When the cells were exposed for longer periods (*h*>12), the viability increased according to treatment time. This result was not unexpected, as it represented not a waning of the effects of the antifungal drugs but the budding of new cells that were unaffected by the drug. The increase in viability was greater in flucytosine treated cells than in amphotericin B treated cells, indicating that *C. albicans* is more sensitive to amphotericin B.

**Figure 2:**
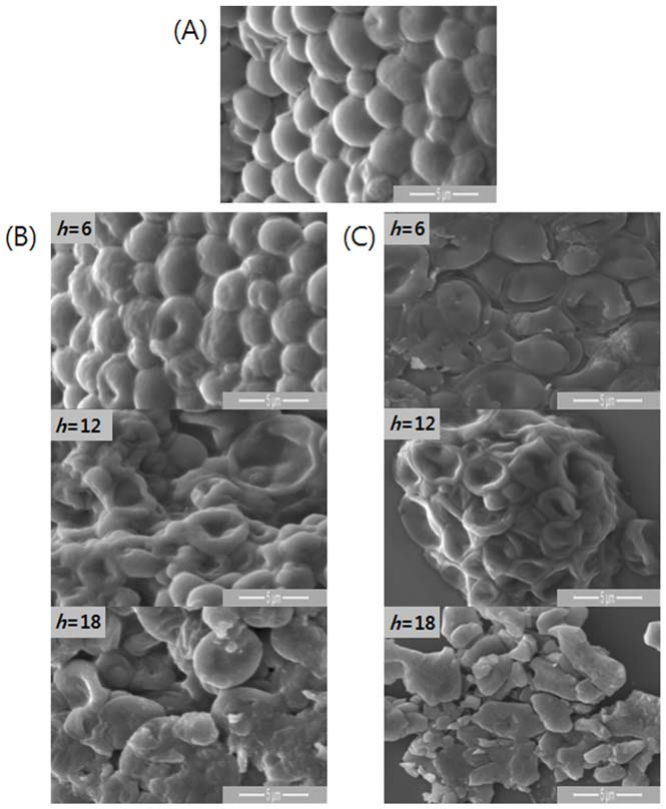
SEM images of *C. albicans* cells. A is the image of untreated cell. B and C show images cells treated with flucytosine (A) and amphotericin B (B) for *h* = 6, 12, and 18, respectively.

SEM images of *C. albicans* cells untreated (A), treated with flucytosine (B) or treated with amphotericin B (C) revealed ultrastructural changes in the *C. albicans* due to the antifungal drugs (Fig. 2). Untreated cells had well defined, intact shapes with smooth surfaces. Cells treated with drugs showed considerable morphological alterations including deformation and shrinkage. In both cases, the cells were significantly destroyed when treated more than 12 hours.

**Figure 3:**
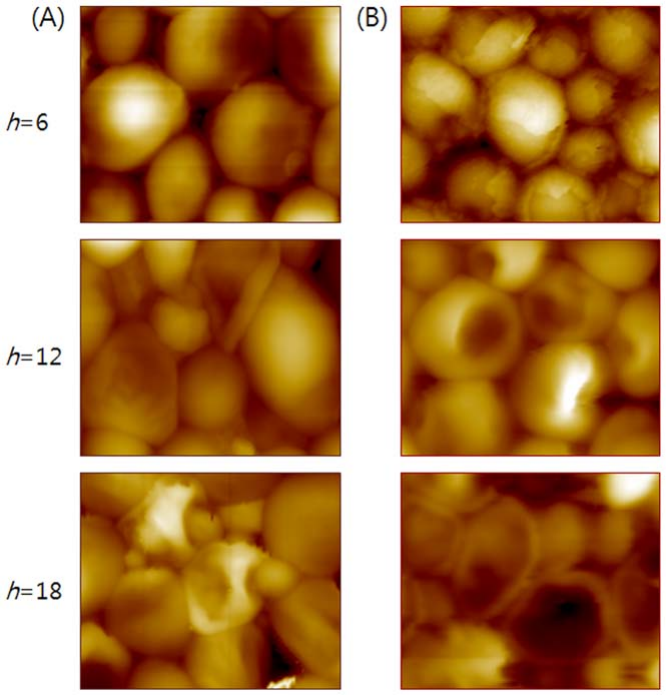
AFM images of *C. albicans* cells. A and B show images of cells treated with flucytosine and amphotericin B for *h* = 6, 12, and 18, respectively.

Representative AFM images of the *C. albicans* treated with flucytosine (A) and amphotericin B (B) are shown in Figure 3. The AFM images clearly show nanoscale morphological changes induced by the antifungal drugs. The most dramatic changes in the membrane of *C. albicans* after treatment with antifungal drugs were observed when the cells were exposed to the drugs for 6 hours. Cells treated with flucytosine were just starting to show collapse of the outer membrane and their shape was becoming irregular compared to the untreated cells (Fig. 3A). In the case of cells treated with amphotericin B (Fig. 3B), the outer membranes were peeling off instead of collapsing. For treatments longer than 12 hours, both cell treatments showed similar changes in the shape and morphology including deformation and shrinkage. These changes are similar to the results observed with SEM.

**Figure 4:**
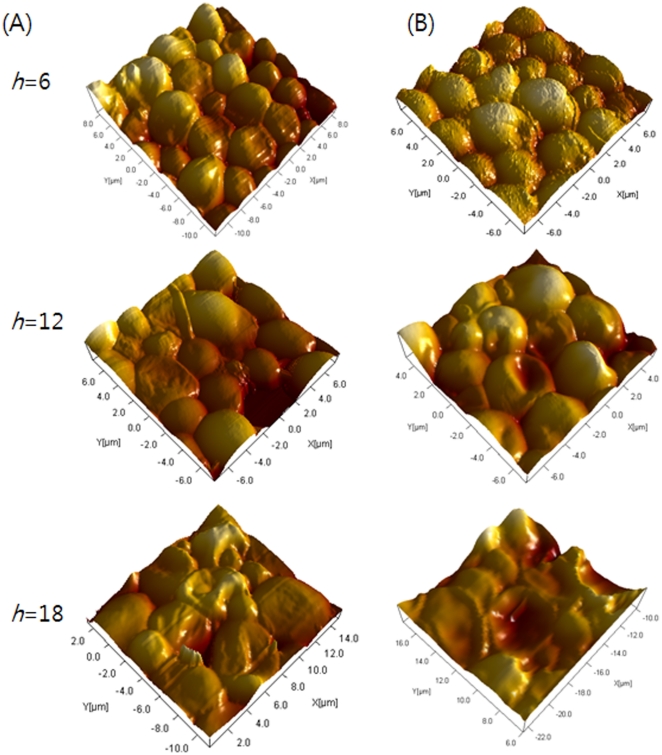
Three-dimensional AFM images. A and B correspond to three-dimensional images of Fig. 3(A) and (B), respectively.

AFM provides a three-dimensional image of the cell surface on a nanometer scale. In the treated cells, the three dimensional structures of *C. albicans* differed in Z axis values according to the drug treatment time. In cells treated with flucytosine, the Z axis values were 240 nm/div, 150 nm/div and 80 nm/div at *h* = 6, 12, and 18, respectively (Fig. 4B). In cells treated with amphotericin B, the values were 300 nm/div, 200 nm/div, and 100 nm/div at *h* = 6, 12, and 18, respectively (Fig. 4B).

**Figure 5:**
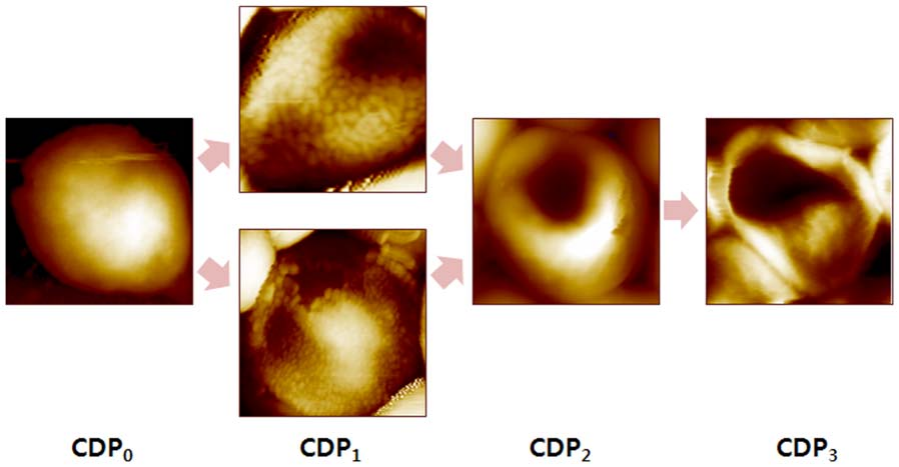
Cell death phase (CDP) of *C. albicans* induced by flucytosine and amphotericin B. The cell death process can be divided by four phase according to the changes in shape and morphological properties.

According to SEM and AFM images, the cell death phase (CDP) of *C. albicans* can be divided into four steps (Fig. 5). The first step is CDP_0_, in which the cells are not visibly affected by the drug and show very clear, intact, and defined shapes. The second step is CDP_1_, in which the cell shape is partially deformed, collapsed or peeled off depending on the type of antifungal drug used. The third step is CDP_2_, in which the cells lose their original shapes and porosity is expanded. The width and depth of the hole is about 50–70% of the cell diameter. The final step is CDP_3_, in which the cells are shrunken and completely destroyed.

**Figure 6:**
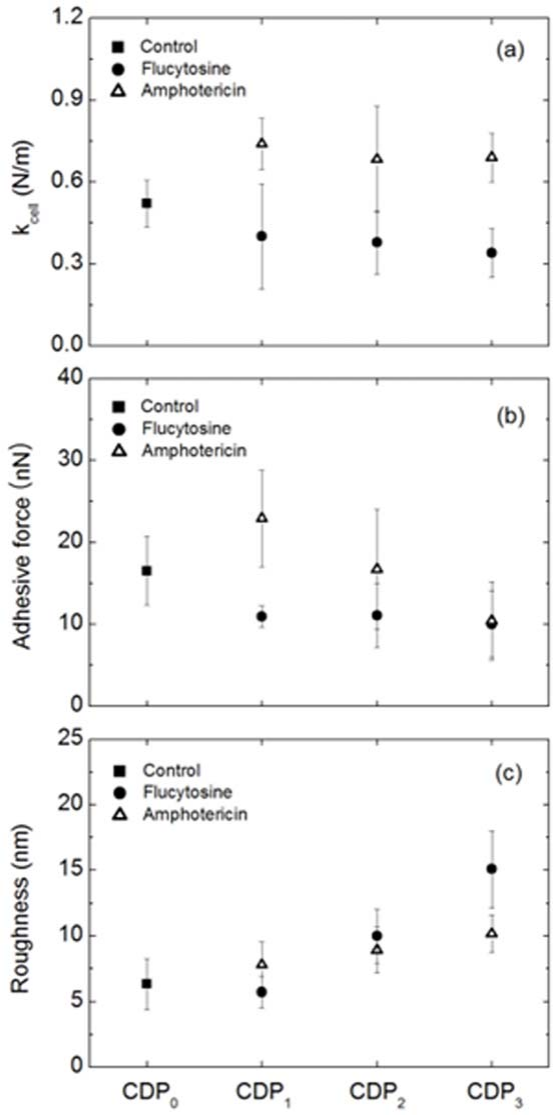
Changes in biophysical properties of *C. albicans* according to CDP. (A) shows changes in the stiffness of cells treated by flucytosine and amphotericin B. (B) and (C) show changes in the adhesive forces and the surface roughness.

The effects of antifungal drugs on the biophysical properties of *C. albicans* were also investigated using AFM measurements. Obvious changes in stiffness, adhesive force, and roughness were observed in both groups of treated cells (Fig. 6), but the changes seen were dependent on the type of antifungal drug used. Compared to untreated cells, the cells treated with flucytosine were softer, while the cells treated with amphotericin B were harder (Fig. 6a). The stiffness did not change with the use of CDP in either case. The adhesive force of cells treated with flucytosine was lower than that of untreated cells (Fig. 6b). The adhesive force of cells treated with amphotericin B was greater than that of the untreated cell at CDP_1_, but decreased to a similar or lower value than CDP_0_ at CDP_2_ and CDP_3_. Since the membrane shrinks as the cell is destroyed, the roughness of the cell surface was increased in both groups of treated cells (Fig. 6c).

## Discussion

Although *C. albicans* is most often associated with candidiasis, the development of a universal therapeutic approach to *C. albicans* infection is still a great challenge because of the vast problems faced with conventional treatments such as toxicity, side effects, resistance, and relapse [3]. The use of natural products and home remedies for the treatment of candidiasis has been gaining popularity both in practical use and in research. Studies of natural drugs showing *in vitro* antifungal activities were first performed by Maruzzella and others in 1958 [9]. Since then, a variety of natural antifungal compounds have been discovered [3], [4], [7], [8], but the mechanisms of action of these natural compounds are not yet fully understood. To understand the antifungal activity of drugs from natural sources, it is necessary to clearly show the death process of *C. albicans* in both quantitative and qualitative terms. Thus, in this work, the CDP of *C. albicans* induced by two commonly used antifungal drugs of flucytosine and amphotericin B was investigated using SEM and AFM by measuring cell viability, and morphological and biophysical properties.

Antifungal agents affect the morphology and physical properties of cells, such as shape, size, height, roughness and stiffness [10]–[13]. Adherence of cells is also affected by antifungal agents. Several works suggested that the activity of antifungal agents can be evaluated by changes in morphology and physical properties of the fungal cells. Using transmission electron microscopy (TEM), SEM, and AFM, morphological changes in *C. albicans* have been investigated qualitatively. TEM is a microscopy technique to use a beam of electrons, which is transmitted through a thin sample and interacted with the sample as it passes through. Therefore, it is usually employed to see inner part of the sample. The SEM images a sample by scanning it with a high-energy beam of electrons, and it is capable of imaging at a significantly higher resolution than light microscopes. However, for SEM imaging, a biological sample should be completely dry, because the sample chamber is at high vacuum. Tyagi *et al.* investigated the antimicrobial activity of lemon grass oil against *C. albicans* observed by *in situ* TEM, SEM, and AFM [10] and determined that *C. albicans* treated with LGO vapor ruptured completely, cells treated with LGO in broth shrank and that the surface roughness of cells treated with LGO was significantly higher in control *C. albicans* than in treated cells. In recent years, it has been increased the preference of AFM in biomaterial imaging since its several advantages over the other spectroscopy. AFM provides not only a two-dimensional image of a sample but also a three-dimensional surface profile. For AFM imaging, the samples do not require any special treatments such as coatings with metal or carbon, and dry. Additionally, the images can be obtained in ambient air or even a liquid environment. Adya *et al.* investigated the toxic effects of ethanol on yeast cell surface morphology using AFM [11]. The cell surface morphological changes were correlated with cellular stress physiology. By exposing yeast to increasingly stressful concentrations of ethanol, cell viabilities and mean cell volumes were decreased.

In this work, the morphological changes of *C. albicans* induced by antifungal drugs were clearly revealed by SEM and AFM. Especially, the dependence of morphological changes on the mechanism of action of antifungal agents was clearly distinguished in the AFM images. As shown in Fig. 3(A), the death process of *C. albicans* treated by flucytosine was accompanied by membrane collapse, expansion of the hole, and shrinkage. These changes can be understood by the mechanism of action of the flucytosine. The flucytosine is a fluorinated pyrimidine analog which mainly acts on RNA and DNA. It shows antifungal activity by inhibiting RNA and fungal replication by DNA synthesis inhibition [14] and is metabolized into 5-fluorouracil. The damaged RNA and DNA prevent a normal metabolism, a protein synthesis, and are accompanied by the destruction of microtubule. According to this mechanism, it thought to be that the interior of the cell is firstly destroyed by the flucytosine and it eventually results the collapses the outer membrane. While the *C. albicans* treated with amphotericin B shows a quite different cell death process as shown in Fig. 3(B). The membranes of cells treated with amphotericin B were firstly peeled off, this exfoliation was observed mainly after 6 hours treatment. By the further treatment, the membrane was holed and shrunk as like the cell treated by flucytosine. These morphological changes also show a good agreement with the mechanism of action of amphotericin B. The Amphotericin B which is antifungal polyene is known to acts on cell walls and membranes even though the precise mechanism still remains unclear [15]. This drug binds to ergosterol, which is the principal sterol in membrane of fungal cell, and then destroys the integrity of the fungal membrane. Fortunately, the very subtle changes in the membrane were detected by AFM.

Physical properties of the cells, such as roughness, stiffness, and adhesive force, were also obtained by using AFM, and the changes induced by antifungal drugs were determined quantitatively. Cells treated with flucytosine were softer than untreated cells, while cells treated with amphotericin B were harder than untreated cells. Although it is not yet fully understood how cell stiffness is regulated, it is known that the stiffness of cell is largely determined by physical property of membrane, cytoskeletal architecture, and the network of intermediate filaments. Because of this fact, the cell stiffness can provide insight into the status and function of the cells. Several studies reported that the cell stiffness changed with the progression of diseases [16], [17]. The change in the cell stiffness observed in this work is related to the cell death caused by antifungal agent. The lowered stiffness in the cell treated with flucytosine can be understood by the mechanism of action of flucytosine. As mentioned before, the flucytosine mainly acts on RNA and DNA synthesis and results in the metabolic disorder. Therefore, the destroyed cytoskeletal architecture or microtubule is responsible for the softness of the membrane. The change in the stiffness was not significantly dependent on the progression of cell death. The larger stiffness in cells treated with amphotericin B is not well understood, however, it clearly shows that the changes in physical property of the cell depend on variety of antifungal agents.

In both cells, it was not observed a significant change in the adhesive force except the cell treated with amphotericin B at CDP_1_. The adhesive force is the strength of the interaction between the cell membrane and AFM tip. The force depends on the sample conditions and dimensions of the tip, which changes with humidity, temperature, and tip diameter [18]. To minimize these problems in this work, all images were measured with the tip manufactured in the same process and the similar environmental conditions. The adhesive force is also very sensitivity to the properties of the sample, such as hydrophobic or hydrophilic because of the AFM tip property. The tip used in this work is made by silicon which is hydrophobic naturally, but the silicon is easily oxidized in air and turns to hydrophilic [19]. The membrane is hydrophobic naturally, and its property does not changed during the cell death process. Therefore, the larger adhesive force at CDP_1_ in the cell treated with amphotericin B may be due to internal secretion occurring after destruction of the cell membrane. Strictly speaking, it is not an adhesive force of the cell, but a force caused by certain sticky substance secreted in the cell.

In conclusion, the effects of two antifungal drugs on *C. albicans* were investigated by SEM and AFM. It was observed the morphological changes according to the treatment conditions, and the changes in the biophysical properties were determined quantitatively. The cell death process was defined based on the changes of morphology and biophysical properties. These findings will be applied to further analysis of antifungal activity of compounds derived from natural sources.

## Materials and Methods

### Culture and preparation of *C. albicans*

*C. albicans* was purchased from the American Type Culture Collection (ATCC, Rockville, MD, USA), maintained in Sabouraud broth, and incubated at 37°C for 24 hours in a shaking incubator at 180 rpm. The samples were centrifuged at 2500 rpm for 15 minutes and washed in calcium-, magnesium-free phosphate-buffered saline (DPBS), and cells were used immediately.

### Viability of *C. albicans* after antifungal treatment

For measurements of antifungal cytotoxicity, *C. albicans* was inoculated at 1×10^6^ cells/ml in Sabouraud broth. *C. albicans* in cell suspension were treated with the minimum inhibitory concentrations (MIC)_90_ of antifungal agents, 1 µg/ml amphotericin B and 1 µg/ml flucytosine. After variable incubation times in a shaking incubator at 180 rpm, *C. albicans* cells were centrifuged at 2500 rpm for 15 minutes and then washed with 0.1 M PBS. For the assessment of cell cytotoxicity, cells were counted using a hemocytometer after stained with trypan blue dye (1∶1) to exclude non-viable cells. The MIC_90_ of each drug was determined according to Graziella et al. [20].

### Scanning electron microscopy (SEM)

For SEM measurements, *Candida* cells were smeared on a conducting layer as a thin film, and the samples were platinum-coated by sputtering. SEM observations were made using a FEI Company W-filament SEM embedded in a Focus Ion Beam. All observations were conducted under the conditions of EHT = 10.00 kV, Current = 0.12 nA, WD = 15 mm.

### Atomic force microscopy (AFM)

The AFM system used in this study was the NANOStation II (Surface Imaging Systems, Herzogenrath, Germany), which consists of an AFM scanner and Zeiss optical microscope (Epiplan 500×). The images of *C. albicans* were measured in contact mode (Budget Sensor, Bulgaria) with a reflex-coated silicon cantilever. The properties of the probe used in imaging were: resonance frequency of 190 kHz (±14 kHz) and force constant of 48 N/m (±1.4 N/m). The images were taken with a resolution of 256×256 pixels and a scan speed of 0.2 line/s.

To perform force-distance (FD) curve measurements, the sample surface was first scanned to determine an appropriate site for the force curve without defects or impurities. Cell elasticity was quantified by the following equations: 1/*k*_cell_ = 1/*k*_effective_−1/*k*_cantilever_, where *k*_effective_ and *k*_cantilever_ was determined from the slope of the linear region of the FD curve for a cell and a clean slide glass, respectively [21].
